# Host Factors Influencing the Retrohoming Pathway of Group II Intron RmInt1, Which Has an Intron-Encoded Protein Naturally Devoid of Endonuclease Activity

**DOI:** 10.1371/journal.pone.0162275

**Published:** 2016-09-02

**Authors:** Rafael Nisa-Martínez, María Dolores Molina-Sánchez, Nicolás Toro

**Affiliations:** Structure, Dynamics and Function of Rhizobacterial Genomes, Department of Soil Microbiology and Symbiotic Systems, Estación Experimental del Zaidín, Consejo Superior de Investigaciones Científicas, Calle Profesor Albareda 1, 18008, Granada, Spain; Keio University, JAPAN

## Abstract

Bacterial group II introns are self-splicing catalytic RNAs and mobile retroelements that have an open reading frame encoding an intron-encoded protein (IEP) with reverse transcriptase (RT) and RNA splicing or maturase activity. Some IEPs carry a DNA endonuclease (En) domain, which is required to cleave the bottom strand downstream from the intron-insertion site for target DNA-primed reverse transcription (TPRT) of the inserted intron RNA. Host factors complete the insertion of the intron. By contrast, the major retrohoming pathway of introns with IEPs naturally lacking endonuclease activity, like the *Sinorhizobium meliloti* intron RmInt1, is thought to involve insertion of the intron RNA into the template for lagging strand DNA synthesis ahead of the replication fork, with possible use of the nascent strand to prime reverse transcription of the intron RNA. The host factors influencing the retrohoming pathway of such introns have not yet been described. Here, we identify key candidates likely to be involved in early and late steps of RmInt1 retrohoming. Some of these host factors are common to En^+^ group II intron retrohoming, but some have different functions. Our results also suggest that the retrohoming process of RmInt1 may be less dependent on the intracellular free Mg^2+^ concentration than those of other group II introns.

## Introduction

Retroelements, such as retroviruses and retrotransposons, are selfish genetic elements that use an RNA intermediate for mobility. Their survival and spread within a particular genome depends on a balance between their reliance on host factor machinery for mobility and their ability to overcome host defense mechanisms [1]. Group II introns are self-splicing catalytic RNAs and mobile retroelements found in organelles (mitochondria and chloroplast) of lower eukaryotes and plants, and in bacteria and archaea genomes [2–7].

Group II introns consist of a structured RNA that folds into six double-helical domains, DI to DVI [8,9]. Unlike organellar introns, most bacterial group II introns have an open reading frame (ORF) in DIV encoding an intron-encoded protein (IEP) with an N-terminal reverse transcriptase (RT) domain, followed by an RNA-binding domain (X domain). In some of these IEPs, the X domain is followed by a C-terminal DNA-binding (D) region and a DNA endonuclease (En) domain. The mobility of group II introns, with and without D and En domains, is mediated by a ribonucleoprotein (RNP) complex consisting of the IEP and the spliced intron lariat RNA that recognizes intron targets through both RNP components [10–12].

The retrohoming pathway of group II introns with IEPs displaying endonuclease activity (e.g. *Lactococcus lactis* intron Ll.LtrB) involves the full reverse splicing of the intron RNA into the top strand of the DNA joining the two exons. The IEP cleaves the bottom strand, usually 9 nucleotides (nts) downstream from the intron-insertion site, and uses the 3’ end of the cleaved DNA strand for target DNA-primed reverse transcription (TPRT) of the inserted intron RNA [6]. Host factors complete the insertion of the intron cDNA and participate in degradation of the intron RNA template strand, intron second strand DNA synthesis, the resection of DNA overhangs, and final ligation [13–16]. By contrast, the major retrohoming pathway of introns with IEPs naturally lacking endonuclease activity (e.g. the *Sinorhizobium meliloti* intron RmInt1) is thought to involve insertion of the intron RNA into the template for lagging strand DNA synthesis ahead of the replication fork, possibly because its mechanism of synthesis is necessary to prime reverse transcription of the intron RNA [17].

For the En^+^ Ll.LtrB intron, initial genetic analyses [13] in the heterologous *E*. *coli* host identified diverse factors influencing intron mobility, by reducing retrohoming efficiency, such as the degradative enzymes RNase I and E, which may act by degrading the intron RNA, and exonuclease III (XthA), which may degrade the newly synthesized cDNA or the top strand in the upstream exon. RNase E has been shown to block retrohoming in cells in particular physiological states [14]. RNase H1 and Pol I (5’-3’exonuclease) may be involved in degrading the inserted intron RNA, whereas *dnaE* Pol III may be involved in second-strand cDNA synthesis, together with other accessory polymerases (Pol II, IV and V). Other exo- and endonucleases [RecJ, MutD (DnaQ), and SbcD] are probably required to resolve intermediates, whereas DNA ligase A probably completes the process by sealing leftover DNA strand nicks.

Other loci potentially influencing retrohoming frequency have been identified. These genes encode proteins involved in RNA processing (*rne*, *pnp* and *trmE*), global transcriptional regulation sensing the nutrient status of the cell (*cyaA* and *SpoT*), energy metabolism (*atpA*, *cyoB* and *cyoE*), replication (helicase *rep*) and a putative transporter (*yidR*). The late steps of Ll.LtrB retromobility appear to be stimulated by starvation, and the global regulators cAMP and ppGpp appear to be involved in controlling the accessibility of the chromosomal DNA target [15].

Recent genetic and biochemical studies by Lambowitz´s group [16] have led to the establishment of a model of host factor function in the retrohoming of Ll.LtrB in *E*. *coli*. RNase H1 degrades the inserted intron RNA template, leaving small fragments that could be used for priming intron second-strand DNA synthesis (top strand). After synthesis of the full-length intron cDNA (bottom strand) and its extension into the 5’exon by the RT domain or a host DNA polymerase, the branched intermediate is recognized by a replication start protein, PriA or PriC, which initiates a replisome-loading cascade of the accessory proteins DnaC and DnaT, and the replicative helicase DnaB, leading to top-strand DNA synthesis by the host-replicative polymerase Pol III. The 5’-3’exonuclease activity of Pol I is probably involved in degrading the remaining small intron RNA fragments attached to the newly synthesized DNA and in gap-filling. The single-stranded DNA-binding protein Ssb and the primase DnaG are also required for top-strand DNA synthesis. DnaG is known to synthesize short RNA primers, which are used by Pol III. Interestingly, DnaG seems to be involved in initiating bottom-strand cDNA synthesis, possibly by copying the 5’ top-strand DNA overhang before the RT domain initiates cDNA synthesis. Other host proteins inhibiting retrohoming *in vivo*, but not *in vitro*, by affecting chromosome structure, DNA replication or transcription, include GyrB, Hns, RpoH, SbcC and Tus. Similarly, MnmE, which is involved in tRNA modification, may also affect intracellular intron RNP levels.

Intracellular free magnesium concentration is also important for the retrohoming of Ll.LtrB and the splicing of yeast mitochondrial group II introns [18–20]. Recent studies have demonstrated that low Mg^2+^ concentration has a negative effect on the mobility of the Ll.LtrB intron [20], and the supplementation of human cell growth media with 80 mM MgCl_2_ is required to promote the retrohoming of Ll.LtrB in this background [21].

The host factors required for the retrohoming of En^-^ introns have yet to be identified. Nevertheless, based on the findings for Ll.LtrB, it has been hypothesized [16] that insertion pathways for introns of this type probably require the stalling of the replication fork and dissociation of the replisome, enabling the RT domain to initiate reverse transcription from the nascent strand, to recruit the replisome again for second-strand DNA synthesis and to continue host DNA replication.

We analyzed the influence of *Sinorhizobium meliloti* host factors on the major retrohoming pathway of the group II intron RmInt1. By screening over 100 mutants of *S*. *meliloti* strains 2011 and 1021 with impairments of various cellular processes potentially related with the retrohoming pathway, we identified major candidates for involvement in the early and late steps of RmInt1 retrohoming. Some of the host factors identified are also involved in En^+^ group II intron retrohoming, but we also identified new factors, and demonstrated an unexpected lower dependence on Mg^2+^ concentration.

## Results and Discussion

### Host factors influencing the retrohoming of RmInt1 *in vivo*

**Fig 1:**
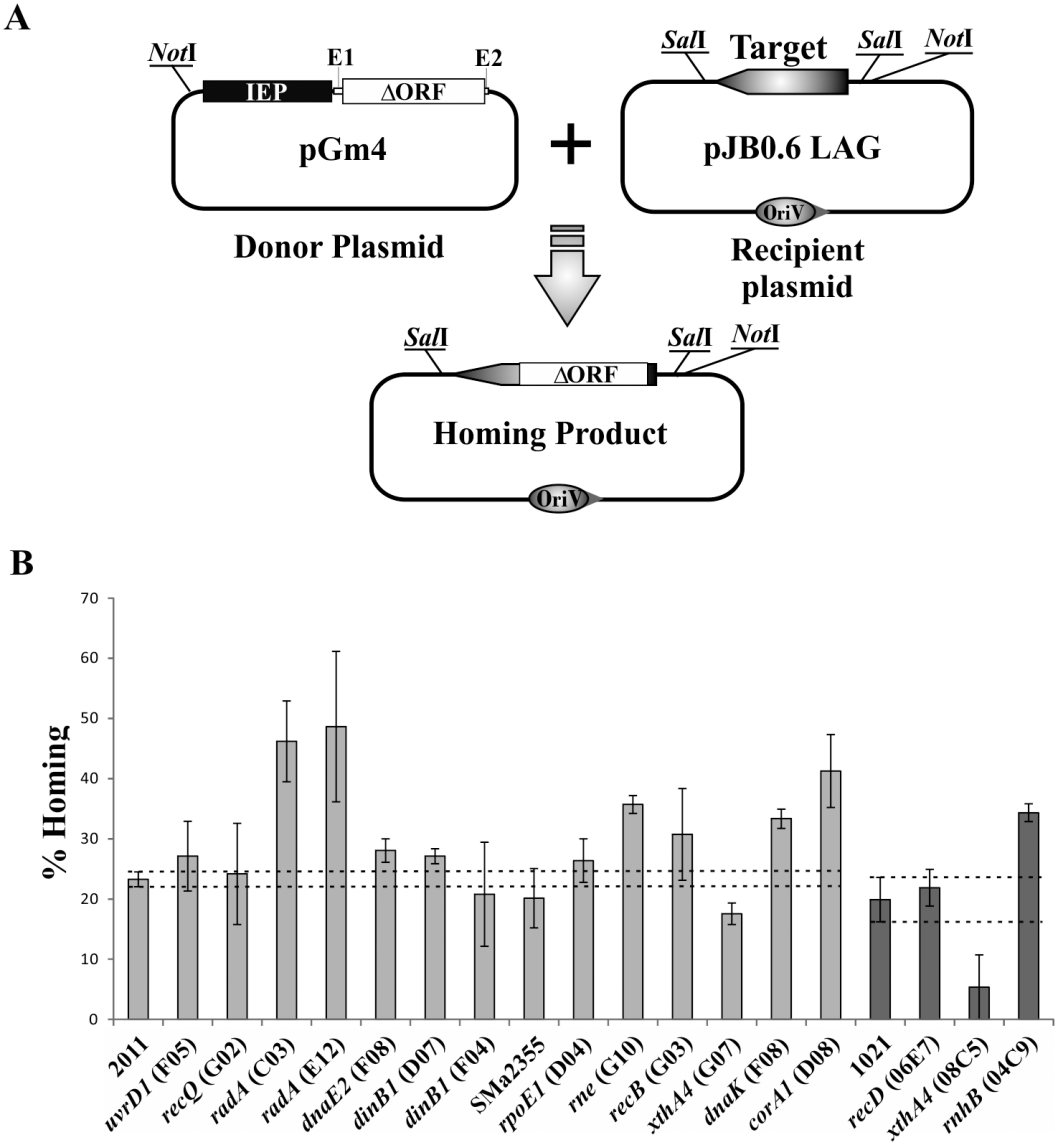
Plasmid to plasmid retrohoming assays. (A) Schema of the double-plasmid retrohoming assay: the target on the recipient plasmid (pJB0.6LAG) could be invaded by an intron from the donor (pGm4), resulting in the homing product. (B) retrohoming efficiencies of the RmInt1-ΔORF in the *S*. *meliloti* wild-type strains (2011 and 1021) and different mutants are plotted. The values correspond to the mean ± SEM for three determinations. Dashed lines indicate the confidence interval of the retrohoming efficiency for RmInt1-ΔORF in the wild-type strains 2011 and 1021 assayed in parallel.

The *in vivo* retrohoming efficiency of RmInt1 in its natural host *S*. *meliloti* was evaluated in a double-plasmid assay (Fig 1A), with an intron donor plasmid expressing the RmInt1 IEP followed by the ΔORF intron RNA, and a recipient plasmid containing the RmInt1 target site (-176/+466) inserted in the correct orientation to serve as the template for lagging-strand synthesis at the DNA replication fork (pJB0.6LAG). This arrangement favors the preferred RmInt1 retrohoming pathway. A donor plasmid for use in kanamycin-resistant mutant strains was required. We therefore constructed pGm4, a new intron donor plasmid conferring gentamycin resistance. The retrohoming efficiency of the ΔORF intron derivative of pGm4 was first evaluated in the intron-less strain *S*. *meliloti* RMO17. The intron donor plasmid pKGEMA4 was used as the reference for retrohoming efficiency (fraction of recipient targets invaded by the intron). The mobility efficiency of the new construct pGm4, in *S*. *meliloti* RMO17, was similar to that of pKGEMA4 (S1 Fig). We therefore considered pGm4 to be suitable for use in the evaluation of RmInt1 retrohoming.

We then determined RmInt1 retrohoming efficiency, in a double-plasmid assay on 105 mutants of *S*. *meliloti* 2011 and 1021 (S1 Table). Wild-type strains were used as controls. Retrohoming efficiency varied between the mutants, the position of the mutation within a particular locus, and between strains. Due to the variability of the retrohoming efficiency test, we selected only those mutants with a retrohoming efficiency preliminary estimated ≤50% or ≥150% relative to the wild type for further studies. We re-evaluated the homing efficiency of 17 selected mutants (14 derived from strain 2011 and 3 from strain 1021; S2 Table) in at least three independent experiments. The results obtained revealed eight mutants (Fig 1B) showing a consistent major effect on the RmInt1 retrohoming efficiency, corresponding to six different loci predicted to be involved in DNA-processing functions (*xthA4*, *radA*, and *dnaK*), RNA-processing functions (*rne* and *rnhb*), and Mg^2+^ transport (*corA1*).

### XthA4 is required for efficient RmInt1 retrohoming

**Fig 2:**
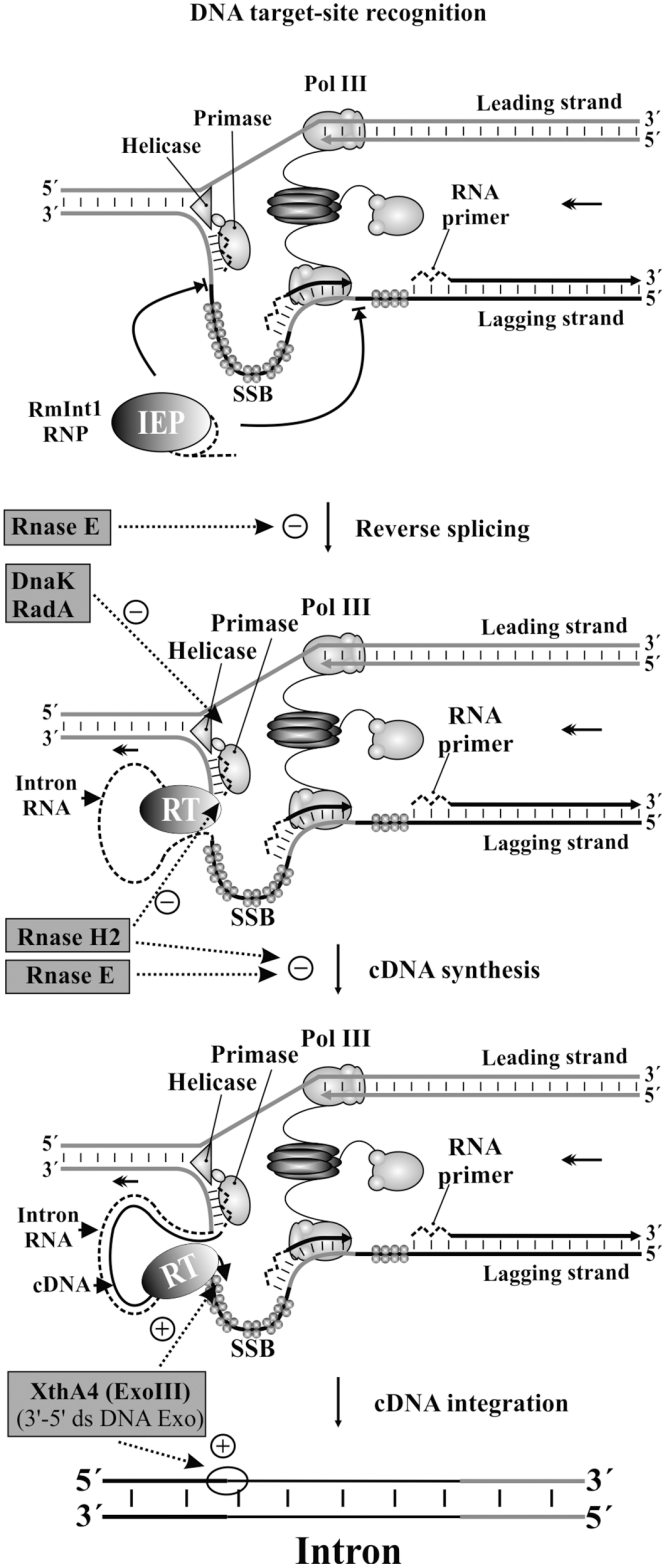
Key host factors accessory functions in the retrohoming pathway of RmInt1. Two headed arrow indicates the replication fork progression. RmInt1 RNP complexes recognize the target site on single-stranded DNA linked to DNA replication, involving invasion of the DNA strand that serves as a template for lagging strand synthesis. The reverse transcription of the inserted intron RNA could be primed by RNA primers (zigzag dashed lines), synthesized by the primase, or partial polymerization of the Okazaki fragments by DNA polymerase III. As an example, we represented the RmInt1 insertion beside the RNA primer. Intron RNA is shown as a dashed line. Enzymes found in this work are highlighted on grey rectangles, and their putative sites of action [facilitatory (+) and inhibitory (-) roles] are indicated by dotted arrows.

The *xthA4* gene encodes a 3’-5’double-stranded DNA exonuclease (ExoIII), and its mutation in both hosts greatly decreases RmInt1 retrohoming efficiency, to about 75% and 27% wild-type levels for strains 2011 and 1021, respectively (Fig 1B and S2 Table). Several catalytic functions have been attributed to this protein in *E*. *coli*: 5’ apurinic/apyrimidinicendonuclease preparing the DNA for subsequent excision, repair synthesis and DNA ligation, 3’-5’ exonuclease/phosphatase activity, 3’-phosphodiesterase activity, and an RNase H-like activity [22]. By contrast to our findings, an earlier study on the group II intron Ll.LtrB described an eight-fold increase in retrohoming frequency in an *xthA1 E*. *coli* mutant [13]. The authors of this previous study suggested that 3’-5’ exonuclease (Exo III) activity led to the degradation of the newly synthesized cDNA, preventing repair, or that this 3’-5’ exonuclease degraded the top strand in the upstream exon and/or competed with Pol III for primers during second-strand synthesis. Our results support that this exonuclease is involved in retrohoming, but, at least for introns lacking the endonuclease domain, it might be required for completion of the process, possibly by degrading the extended cDNA in the 5’exon and allowing the DNA polymerases to fill the gap (Fig 2).

### Host factors with inhibitory functions in retrohoming

Cellular RNases, particularly those constituting the degradosome or alternative degradation complexes, have been identified as potentially involved in the major host defense mechanism driving the inhibition of retrohoming [16]. We found that the retrohoming efficiency of RmInt1 in an RNase E (*rne*) mutant was 153% wild-type levels (Fig 1B and S2 Table). This enhanced intron mobility suggests that RNase E has a negative effect on RmInt1 mobility, probably degrading the intron RNA just before reverse splicing or cDNA synthesis (Fig 2), as described for Ll.LtrB [13,14].

RNase H1 (*rnhA*) seems to be a primordial retrohoming factor for Ll.LtrB. Its absence leads to a significant decrease in retrohoming efficiency, whereas the absence of RNase H2 (*rnhB*) has only a minor effect [13].The significant influence of RNase H1 on the retrohoming process (its absence leading to 11–15% wild-type levels of retrohoming) was subsequently confirmed in other studies [16]. It has been suggested that RNase H1 may be involved in the degradation of the inserted intron RNA template strand, possibly leaving residual RNA fragments behind that might serve as primers for the synthesis of the second-strand DNA complementary to the intron cDNA [16]. In *S*. *meliloti* species, RNase H1 does not appear to play a major role in retrohoming efficiency, hence it is possible that other enzymes able to eliminate RNA/DNA hybrids also contribute to the removal of inserted intron RNA. However, by contrast to what was reported for Ll.LtrB, we observed a substantial increase in retrohoming rates (Fig 1B) in an *S*. *meliloti* RNase H2 (*rnhB*) mutant (~172% wild-type levels, S2 Table). Both RNase H1 and RNase H2 can eliminate RNA/DNA hybrids, but, in some biological systems, RNase H2 is essential for the removal of single ribonucleotides covalently attached to DNA during genome duplication (primers for lagging strand synthesis) and may have specific access to the R-loop during replication [23,24]. This postulated interaction of RNase H2 with the replication and repair machineries may account for its role in impeding RmInt1 retrohoming, a process associated with replication that is probably dependent on primers for lagging strand synthesis (Fig 2).

The efficiency of retrohoming was higher (143% wild-type levels) in the absence of the heat-shock chaperone protein DnaK (Fig 1B and S2 Table). It has been proposed that the DnaKJ chaperone remodels the replisome to facilitate repair, whereas DnaK controls the template switch repair process at the replication fork [25]. DnaK is also required to release the DnaB helicase, which is tightly associated with the lambda P protein during replication of the DNA of bacteriophage λ [26]. *dnaK* mutants are sensitive to replication fork damage and accumulate large amounts of single-stranded DNA, as demonstrated by the constitutive induction of the SOS response. As RmInt1 preferentially inserts into single-stranded DNA due to the absence of endonuclease activity, increases in retrohoming efficiency in this mutant may be related to the accumulation of ssDNA at the replication fork (Fig 2).

The two *radA* mutants analyzed had retrohoming rates twice those in the wild type (Fig 1B and S2 Table). RadA is a repair protein involved in DNA replication and repair [27–30]. The effects of the RadA and DnaK proteins in RmInt1 retrohoming may be correlated (Fig 2), because single-stranded DNA accumulates in both mutants [27].

### Effect of magnesium concentration on the *in vitro* splicing and the *in vivo* RmInt1 retrohoming pathway

We found that RmInt1 retrohoming rates were much higher than wild-type rates (177%) in the Mg^2+^ transporter mutant 2.03.D08 *corA1* (Fig 1B and S2 Table). The increase was particularly remarkable, as it has been shown that low intracellular free Mg^2+^ concentration leads to lower levels of retrohoming for the *L*. *lactis* group II intron Ll.LtrB [20]. These findings led us to investigate in more detail the effect of magnesium concentration on the *in vitro* splicing and the RmInt1 retrohoming pathway *in vivo*.

**Fig 3:**
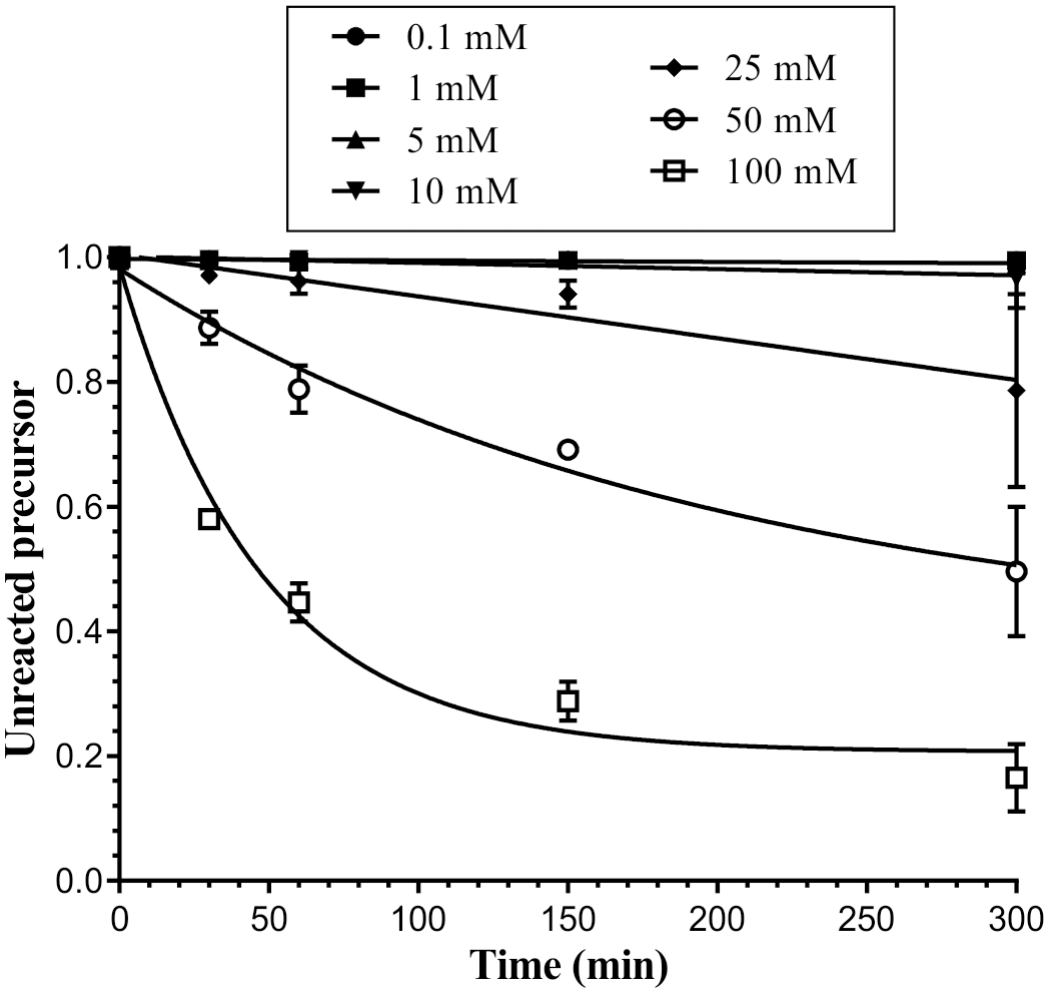
Dependence of *in vitro* RNA splicing of the RmInt1 intron on Mg^2+^ concentration. RmInt1 ΔORF-WT self-splicing was evaluated at 50°C in a reaction buffer supplemented with 80 mM MOPS, 0.5 mM NH_4_(SO_4_)_2_ and the MgCl_2_ concentrations indicated at the top of the plot. Products were analyzed as described in the Materials and Methods. The graph shows the fraction of precursor molecules not yet reacted over time. A single exponential curve was fitted to the data with GraphPad Prism 6 software.

The self-splicing of group II introns is known to be strongly linked to the presence of Mg^2+^ in the reaction mixture [31]. Introns differ in their requirements for divalent cations for *in vitro* splicing. For instance, the *P*. *litoralis* intron can self-splice at magnesium concentrations as low as 0.1 mM MgCl_2_ (20% splicing), although its optimal magnesium concentration is 10 mM [32]. By contrast, the *L*. *lactis* Ll.LtrB intron requires 50 mM MgCl_2_ for efficient *in vitro* processing [33]. We investigated the effect of Mg^2+^ concentration on RmInt1 splicing *in vitro*, by modifying the amount of MgCl_2_ added to the reaction mixture and analyzing the splicing products (Fig 3). We performed self-splicing reactions in the presence of 0.1, 1, 5, 10, 25, 50, and 100 mM MgCl_2_, analyzing the reaction at various time points (0, 30, 60, 150, and 300 minutes). Like that of the Ll.LtrB intron, the splicing reaction of RmInt1 was dependent on Mg^2+^ concentration, with excision products detected only at concentrations of MgCl_2_ exceeding 25 mM.

**Fig 4:**
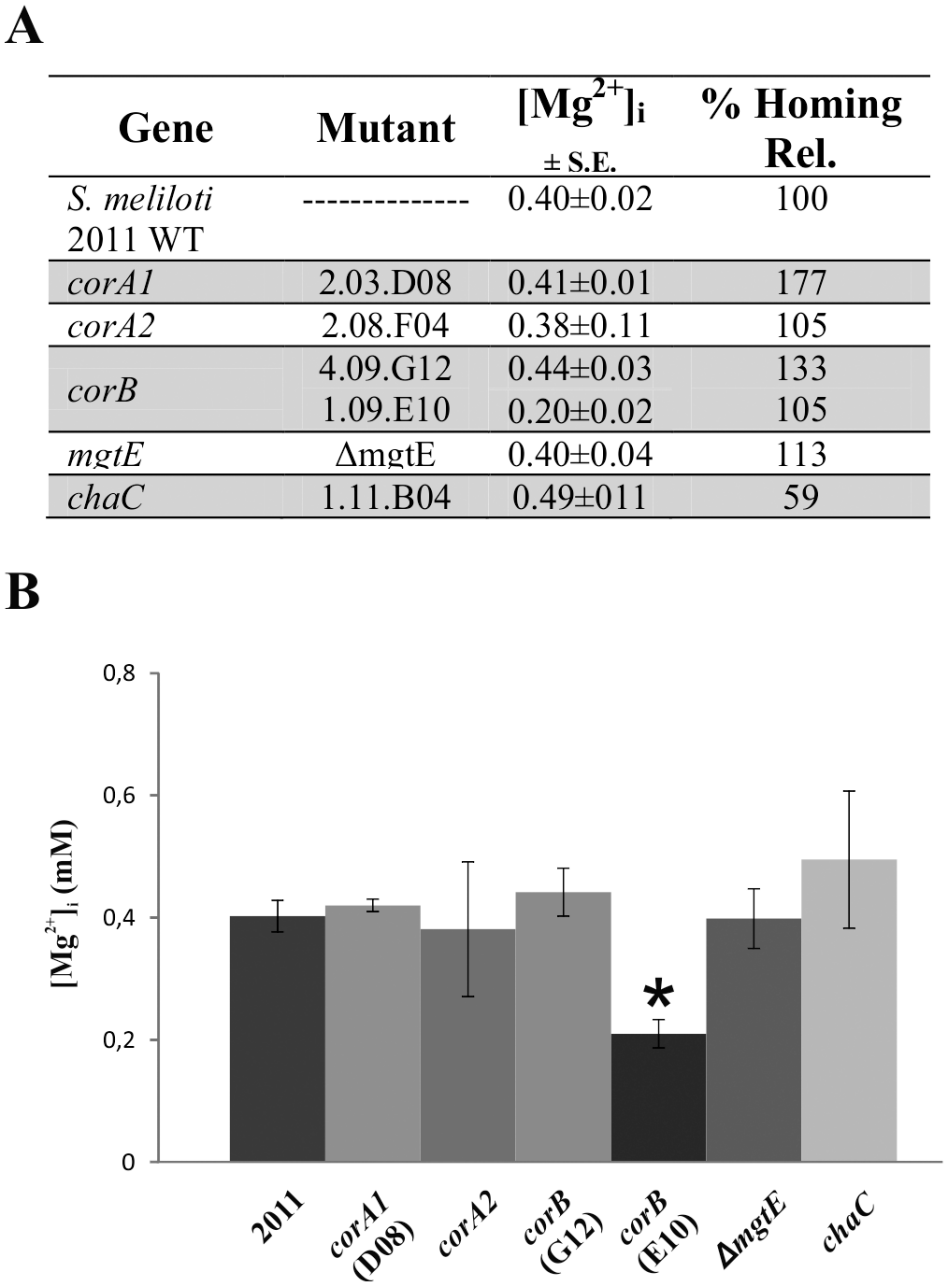
Intracellular free Mg^2+^ concentrations and retrohoming efficiencies of the mutants in different cation transporters. (A) Intracellular free Mg^2+^ concentrations ([Mg^2+^]_i_) of the *S*. *meliloti* 2011 wild-type strain and mutants for various magnesium transporters are shown, as well as their relative homing efficiency. (B) Intracellular free Mg^2+^ concentration are plotted.* indicates the bar corresponding to the *corB* mutant in which [Mg^2+^] was 50% lower than the wild type.

Little is known about magnesium transport in rhizobia, but studies with a *Rhizobium leguminosarum* mini-Tn5 mutation of a gene displaying a high degree of identity to an MgtE-like magnesium transporter suggested that Mg^2+^ transport was correlated with nitrogen fixation [34].The published genome sequence for *S*. *meliloti* strain 2011 includes several genes annotated as transporters of Mg^2+^ or other cations: SMc00399 (*corA1*), SMc00874 (*corA2*), SMc00697 (*corB*), SMc01261 (*mgtE*) and SMc00713 (*chaC*). As indicated above, the retrohoming efficiency in one of the *corA1* mutants was 177% wild-type levels, but intracellular Mg^2+^ concentration (Fig 4B) was found to be similar in this mutant and the wild-type strain (0.41 and 0.40 mM, respectively). Interestingly, homing efficiency in the *chaC* mutant was 59% wild-type levels (Fig 4A), but intracellular Mg^2+^ concentration (Fig 4B) in this disruptant was in the same range as that of the wild type (0.49 mM). Thus, the opposite effect on retrohoming efficiency observed in the *corA1* and *chaC* mutants did not appear to be related to overall intracellular free Mg^2+^ concentration, hence it is possible that these mutations cause differences in the local Mg^2+^ concentration in the regions in which retrohoming occurs that could account for the observed variation in retrohoming efficiency.

For mutants of most of the other Mg^2+^ transporters analyzed, Mg^2+^ concentration was between 0.38 and 0.44 mM, similar to that in the wild type (Fig 4), and retrohoming efficiency was close to that in the wild type (105% to 133%). However, the *corB* mutant with the mini-Tn5 insertion near the 5’ end of the gene (1.09.E10) had an intracellular free Mg^2+^ concentration that was only half that in the wild type and show wild-type retrohoming efficiency (Fig 4). For the Ll.LtrB intron in *E*. *coli* cells [13] halving the intracellular free Mg^2+^ concentration strongly decreases retrohoming efficiency (50% wild-type levels in a *corA* disruptant and 0.5% wild-type levels in an *mgtA* mutant). Our results therefore suggest that the retrohoming efficiency of RmInt1 *in vivo* may be less affected by low Mg^2+^ concentration than that of the Ll.LtrB intron.

## Conclusions

We have identified key candidates likely to be involved in early and late steps of RmInt1 retrohoming. Some of these host factors are common to En^+^ group II intron retrohoming (e.g., RNase E), but some have different functions. Our results suggest that the exonuclease XthA4 might be required for completion of the retrohoming for introns lacking the endonuclease domain, unlike En^+^ introns in which this function (3’-5’ exonuclease) has an inhibitory effect. We also found other host factors (e.g., DnaK and RadA), which functions are likely related to the insertion of the RmInt1 intron RNA into the template for lagging strand DNA synthesis likely by facilitating the accessibility of the target as single-stranded DNA at the replication fork. In contrast to Ll.LtrB intron, we found that RNase H2 has an important role for RmInt1 retrohoming, which may be related to the accessibility to primers for lagging strand synthesis during genome duplication. Finally, we found that the retrohoming process of RmInt1 may be less dependent on the intracellular free Mg^2+^ concentration than those of other group II introns.

## Materials and Methods

### Bacterial strains and growth conditions

The *S*. *meliloti* strains used in this work were RMO17 [35], and the *S*. *meliloti* SU47 derivatives, 1021 and 2011 [36]. *E*. *coli* DH5α was used for cloning and plasmid construct maintenance; *E*. *coli* HB101 (containing the helper plasmid pRK2013) was used for conjugations; and *E*. *coli* HMS174 was used as an external control for the determination of intracellular free magnesium concentration. Rhizobial strains were routinely grown at 28°C in tryptone yeast extract (TY) complex medium [37] or defined minimal medium (MM) [38] and *E*. *coli* was grown at 37°C in Luria–Bertani (LB) medium [39]. Media were supplemented, when required, with antibiotics at the following concentrations: kanamycin (200 μg/ml for *S*. *meliloti* and 50 μg/ml for *E*. *coli*); gentamicin (50 μg/ml for *S*. *meliloti* and 10 μg/ml for *E coli*); tetracycline (10 μg/ml); ampicillin (200 μg/ml); streptomycin (600 μg/ml) and nalidixic acid (10 μg/ml) for *S*. *meliloti*.

### Selection of the *S*. *meliloti* 2011 and 1021 mutants

We investigated the role of host factors in the RmInt1 retrohoming pathway, using mutants obtained from libraries generated with signature-tagged mini-Tn*5* transposons in *S*. *meliloti* strain 2011 and by plasmid integration mutagenesis in *S*. *meliloti* strain 1021 [40–42]. These mutants, listed in S1 Table, were selected on the basis of the involvement of the proteins concerned in host functions and possible involvement in intron mobility: ligases, helicases, DNA repair proteins, polymerase-related proteins, nucleases, chaperones, and cation transporters.

### Construction of the *S*. *meliloti* 2011 Δ*mgtE* mutant

The *S*. *meliloti* 2011 Δ*mgtE* strain was obtained through a double-crossover strategy, using the pK18MobSacB system [43], in which the *mgtE* gene was replaced with the spectinomycin resistance cassette from the pHP45OmSpc vector [44]. A region of about 2900 bp containing the *mgtE* gene, flanked by 700 nts, was amplified from *S*. *meliloti* 2011 genomic DNA with the primers FlmgtEfw (5’-GCGCATGCTTTCGAACCGCTTGACGGC-3’) and FlmgtErv (5’-GGTCTAGAGTTCGAGCGATATTGCCG-3’). This fragment was inserted into the pGEMT easy vector (Promega) and sequenced. The resulting plasmid was used as a template for inverse PCR with the primers InvmgtE5 (5’-GGAAGCTTGGACCCGCCCCTCCAGAGATTA-3’) and InvmgtE3 (5’-GGAAGCTTGCTGCGACGCAAGCGGGC-3’), to delete the *mgtE* gene and added a *Hin*dIII cloning site for insertion of the spectinomycin resistance cassette from pHP45OmSpc. The flanking regions of *mgtE*, including the spectinomycin cassette, were inserted into pK18MobSacB as a *Sph*I-*Xba*I fragment, to yield pK18MSΔmgtE. This construct was used to obtain the *S*. *meliloti* 2011 Δ*mgtE* strain by double crossover. *S*. *meliloti* strain 2011 was transformed with pK18MSΔmgtE, by triparental conjugation. The single crossover strain was first selected on the basis of kanamycin/spectinomycin resistance and sucrose sensitivity. Double-crossover events generated bacteria displaying spectinomycin/sucrose resistance and kanamycin sensitivity. The mutations generated were checked by PCR and Southern blotting.

### *In vivo* homing assays

We determined the *in vivo* homing efficiency of RmInt1 in *S*. *meliloti* wild-type and mutant strains, in a double-plasmid assay, with donor and recipient plasmids. The *S*. *meliloti* mutants were kanamycin-resistant. We therefore constructed an intron expression vector (pGm4) containing a ΔORF derivative in which the sequence encoding the IEP protein was located upstream from the 5’-exon on the plasmid conferring gentamycin resistance. The pGm4 vector was constructed by ligating the 0.8 kb *Sac*I fragment from pKGEMA4 [45] into the *Sac*I site of pBBR1MCS-5 [46] in the same orientation as the T3 promoter. The *Sal*I-*Xho*I fragment from pKGEMA4, containing the constitutive kanamycin resistance gene promoter and the IEP coding sequence, was then ligated between the corresponding sites in pGm4.

We used pJB0.6LAG as the recipient plasmid and pJBΔ129 as the negative control [17]. Donor and recipient plasmids were transferred sequentially to *S*. *meliloti* by triparental conjugation. The *S*. *meliloti* RMO17 transconjugants were selected on MM medium supplemented with kanamycin or gentamicin for donor plasmids, and ampicillin for recipient plasmids. *S*. *meliloti* wild-type 1021 and 2011 transconjugants were selected on TY medium supplemented with gentamicin, ampicillin, streptomycin and nalidixic acid, whereas the *S*. *meliloti* mutants were selected on medium containing all these antibiotics plus kanamycin. Retrohoming events were detected by the Southern hybridization of *Sal*I-*Not*I-digested plasmid DNA with a digoxigenin (DIG)-labeled probe specific for IS*Rm*2011-2, as previously described [47]. The intensity of the bands was determined by densitometry with Image Quantity One software (a Bio-Rad system software package).

Homing efficiency was calculated as the ratio of homing product (H) to the homing product (H) plus non-invaded recipient plasmid (R), expressed as a percentage: [(H /H + R) x 100]. These data were expressed relative to homing rates for the wild-type strain. Retrohoming rates were obtained by analyzing a mixture of plasmid DNA from six to 10 independent transconjugants selected at random. After this first screening, mutants with a homing efficiency ≤ 50% or ≥ 150% were selected for further analysis. Retrohoming assays were carried out at least three times, to ensure that the results were consistent, with individual analysis of plasmid DNA from six to 10 transconjugants.

### Self-splicing assays

Self-splicing assays were carried out with *in vitro* transcripts derived from *Nde*I-digested ΔORF-WT template [48]. Transcription was performed with 4 units of T7 RNA polymerase and 4 μg of linearized plasmid in a final volume of 50 μl containing 1x transcription buffer [10x transcription buffer: 150 mM MgCl_2_, 400 mM Tris-HCl, pH 7.5, 20 mM spermidine and 50 mM DTT], 0.96 mM ATP, 0.96 mM CTP, 0.96 mM UTP, 0.064 mM GTP, 50 μCi [α-^32^P]GTP (3000 Ci/mmol; 10 mCi/ml; Perkin Elmer), 10 mM DTT and 80 U RNAseOUT (Invitrogen) for 2.5 hours at 37°C. Gel-purified transcripts were incubated in 80 mM MOPS buffer pH 7.5 at 95°C for one minute, and then cooled at room temperature for one minute. We added 500 mM (NH_4_)_2_SO_4_ (final concentration) to the RNA samples, and reactions were initiated by adding various Mg^2+^ concentrations, from 0.1 to 100 mM MgCl_2_ (final concentration), as indicated. Samples were incubated at 50°C for 5 hours, with aliquots removed at 0, 30, 60, 150 and 300 minutes. The reaction was stopped by adding quenching buffer (1.8% sucrose, 1x TBE [Tris-borate EDTA, pH 8.3]), 0.018% xylene cyanol dye, 36% (v/v) formamide, and 25 mM EDTA) and placing the mixture on ice. Reaction products were resolved by denaturing electrophoresis in 5% polyacrylamide gels. The gels were dried, and the bands were quantified by densitometry, with Quantity One software (Bio-Rad). Fractions of unreacted precursor molecules were determined from the molar contribution of all intron-containing forms. Molar fractions of self-splicing products were estimated from measurements of radioactivity corrected for the number of uridine residues. The time course data were fitted with one-phase exponential decay curves, with GraphPad Prism software version 6.05.

### Measurement of intracellular free [Mg^2+^]

The intracellular free magnesium ion concentration ([Mg^2+^]_i_) was determined with the acetomethyl ester form of mag-fura-2 (Molecular Probes) and the dispersant Pluronic F-127 (Life Technologies), essentially as previously described [20,49]. We determined [Mg^2+^]_i_ with ~ 1x10^9^ cells from an exponentially growing cell culture (OD_600_ 0.5–0.6) that had been washed once with 0.1% (wt/vol) N-lauroylsarcosine sodium salt solution in 1x TE. We then washed the cells twice in 0.9% NaCl and resuspended them in 1 ml of 0.9% NaCl, 10 mM Hepes (pH7.5). After probe loading, the cells were incubated for 90 minutes at 28°C and washed three times with 0.9% NaCl. Fluorescence data were obtained with a spectrofluorometer QuantaMaster^™^ QM-4 (PTI^®^ Photon Technology International, Lawrenceville, NJ, USA), with stirring of the suspension, at 25°C. Cells without probe loading were used for background subtraction. [Mg^2+^]_i_ values were obtained from the ratio of cellular fluorescence at 509 nm after excitation at 340/380 nm, formulas according to the formula [20,50]:

$${\left[Mg^{2+}\right]}_{i}=K_{d}x\frac{F_{0}}{F_{s}}x\frac{R-R_{min}V_{f}}{R_{min}V_{f}-R},$$

where K_d_ is the dissociation constant of mag-fura-2 AM for Mg^2+^ (1.9); F_0_ and F_s_ are the fluorescence correspond to excitation at 380 nm in 0 mM Mg^2+^ (after adding 1 mM EDTA) and at saturating Mg^2+^ concentration (adding 40 mM Mg^2+^). R is the ratio of fluorescence at 340 and 380 nm. Internal calibration was achieved by incubating the cells with lysozyme (0.5 mg/ml for 10 minutes at room temperature; Roche Life Science) and then adding 1 mM EDTA (R_min_) and 40 mM Mg^2+^ (R_max_), and lysing the cells by adding 1% SDS. The fluorescence ratio (340/380) was then calculated. V_f_ corresponds to a constant (0.85) correcting for the effect of viscosity on fluorescence intensity [50]. Measurements were obtained in four independent experiments, and the data presented are the mean values and standard error.

## Supporting Information

S1 Fig Homing efficiency of pKGEMA4 and pGm4 on *S*. *meliloti* RMO17. (TIF)

S1 Table Mutants of *S*. *meliloti* 2011 and 1021 used in this study. In the first column listed the loci studied in this work and the genome coordinates, according to GenDB browser (http://www.cebitec.uni-bielefeld.de/CeBiTec/rhizogate). The second column indicates the probable function of the genes. The third column corresponds to the mutant used to perform the retrohoming assay. The fourth column indicates the position of the insertion of the mTn5 in 2011 mutants, the plasmid in 1021 mutants or the Spectinomicyn resistance cassette in case of ΔMgtE.
(DOCX)

S2 Table Selected mutants to continue the study. In the first column are listed the wild type strains used in this study, the loci and the genome coordinates, according to GenDB browser (http://www.cebitec.uni-bielefeld.de/CeBiTec/rhizogate). The second column indicates the probable function of the genes. The third column corresponds to the mutant used to perform the retrohoming assay. The fourth column indicates the position of the insertion of the mTn5 in 2011 mutants or the plasmid in 1021 mutants. The fifth column shows the retrohoming efficiency (% ± S.E.M.).The last column indicates the homing efficiency relativized to the wild type (*S*. *melilo*ti 2011 or 1021).
(DOCX)
