# Supplementary figures and images for: Host Factors Influencing the Retrohoming Pathway of Group II Intron RmInt1, Which Has an Intron-Encoded Protein Naturally Devoid of Endonuclease Activity
Source: PLoS One. 2016 Sep 2;11(9):e0162275. doi: 10.1371/journal.pone.0162275 (PMC5010178; doi:10.1371/journal.pone.0162275)

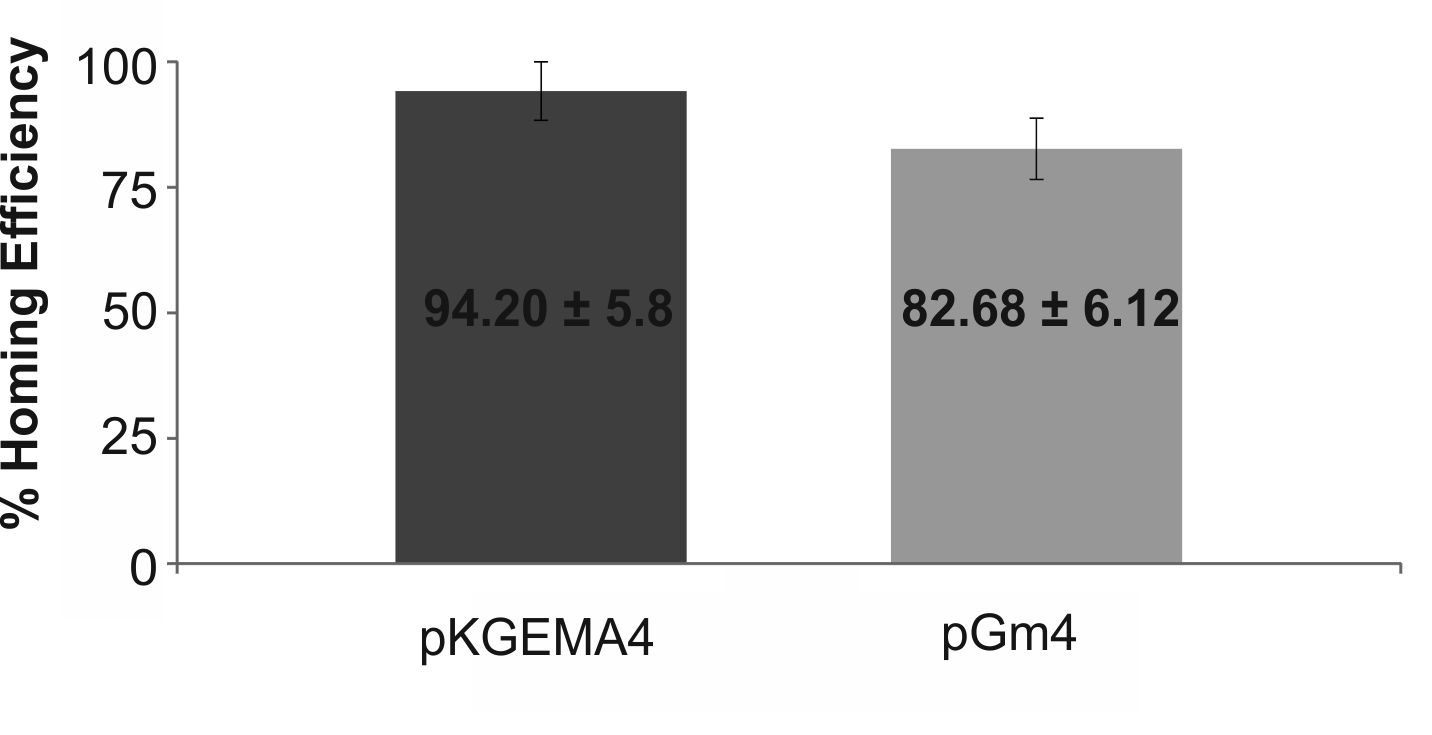

Supplement: S1 Fig — (TIF) [file pone.0162275.s001.tif]
