# Supplementary material for: Host Factors Influencing the Retrohoming Pathway of Group II Intron RmInt1, Which Has an Intron-Encoded Protein Naturally Devoid of Endonuclease Activity
Source: PLoS One. 2016 Sep 2;11(9):e0162275. doi: 10.1371/journal.pone.0162275 (PMC5010178; doi:10.1371/journal.pone.0162275)
Supplement: S1 Table — In the first column listed the loci studied in this work and the genome coordinates, according to GenDB browser (http://www.cebitec.uni-bielefeld.de/CeBiTec/rhizogate). The second column indicates the probable function of the genes. The third column corresponds to the mutant used to perform the retrohoming assay. The fourth column indicates the position of the insertion of the mTn5 in 2011 mutants, the plasmid in 1021 mutants or the Spectinomicyn resistance cassette in case of ΔMgtE. (DOCX) [file pone.0162275.s002.docx]

**S1 Table**

| **Locus**  **(Genome position)** | **Probable Function (*gene*)** | **Mutant** | **Mutation Position** |
| --- | --- | --- | --- |
| **Ligases** |  |  |  |
| **SMa0424**  (230330-229293) | Putative DNA ligase | **2011**mTn5STM.1.01.G09 | 229373- 229374 |
| **SMb21044**  (665732- 663825) | Putative ATP-dependent DNA ligase | **2011**mTn5STM.3.02.F10 | 664637- 664638 |
| **SMb20912**  (1320904- 1319813) | Putative ATP-dependent DNA ligase | **2011**mTn5STM.3.10.E09 | 1319975-1319976 |
| **SMb20686**  (1489827- 1490708) | Putative ATP-dependent DNA ligase | SmPI_**1021.**12.08C2  **2011**mTn5STM.2.11.C07 | 1490183-1490184  1490319-1490320 |
| **SMc03835**  (3501745- 3501161) | 2'-5' RNA ligase: **(*ligT*)** | **2011**mTn5STM.1.07.E12  **2011**mTn5STM.2.07.C01 | 3501736-3501737  3501405-3501406 |
| **Helicases** |  |  |  |
| **SMc00228**  (1791477-1793582) | Probable ATP-dependent DNA helicase: **(**r***ecG*)** | **2011**mTn5STM.3.10.D01 | 1791486-1791487 |
| **SMc01461**  (2306850-2309423) | 3’-5’ RNA/DNA hybrids helicase: (***uvrD1***) | **2011**mTn5STM.2.03.F05  SmPI_1021.12.01H5 | 2307492-2307493  2308993-2308994 |
| **SMc01566**  (2508771-2506861) | ATP-dep 3’-5’ DNA helicase: **(*recQ***) | **2011**mTn5STM.3.02.G02 | 2507083-2507082 |
| **SMc02586**  (77448- 74989) | ATP-dep. helicase: **(*helO*)** | **2011**mTn5STM.2.03.B09 | 77299- 77300 |
| **SMc01090**  (469665- 467776) | ATP-dep. RNA helicase: **(*deaD*)** | **2011**mTn5STM.1.06.H02  **2011**mTn5STM.3.04.B07 | 469071- 469072 468014- 468015 |
| **SMc02152**  (560199- 556690 | Helicase-DNA-binding protein | **2011**mTn5STM.1.04.D01 | 558440- 558439 |
| **SMc02237**  (603400**-** 600854) | ATP-dep. helicase | **2011**mTn5STM.1.06.F08 | 602008- 602009 |
| **Repair protein** |  |  |  |
| **SMc00556**  (1227431- 1225971) | DNA repair protein: **(*radA*)** | **2011**mTn5STM.2.02.C03 **2011**mTn5STM.4.04.E12 **2011**mTn5STM.1.14.F08 | 1226930- 226931 1226787-1226788 1226170-1226171 |
| **SMc01235**  (1689506- 1686588) | Excinuclease ABC subunit A (DNA repair ATP-binding): **(*uvrA)*** | **2011**mTn5STM.4.03.A06  **2011**mTn5STM.1.09.G09 | 1689490-1689489  1687233-1687234 |
| **SMc01236**  (1686495- 1685692) | Conserved hypothetical protein | **2011**mTn5STM.2.12.B04 | 1686489-1686488 |
| **SMc01877**  (2331847- 2330177) | DNA repair protein: **(*recN*)** | **2011**mTn5STM.1.02.G07 | 2330305-2330306 |
| **Polymerase and related proteins.** |  |  |  |
| **SMc03788**  (3455860-3452510) | DNA polymerase III alpha chain  Major replicative enzyme  (DNA Pol III): **(*dna*E2)** | **2011**mTn5STM.5.12.C02  **2011**mTn5STM.2.04.G08  **2011**mTn5STM.3.02.F01  **2011**mTn5STM.4.13.F08  **2011**mTn5STM.3.09.F02  **2011**mTn5STM.4.02.F11  **2011**mTn5STM.1.02.B04  SmPI_**1021**.12.03H10 | 3453150-3453149  3453492-3453491  3452935-3452934  3452877-3452878  3455685-3455684  3453248-3453249  3453643-3453644  3453649-3453648 |
| **SMa0892**  (497392-500655) | DNA polymerase III alpha chain  Major replicative enzyme  (DNA Pol III): **(*dna*E3)** | **2011**mTn5STM.3.11.G05  **2011**mTn5STM.2.02.E09  **2011**mTn5STM.5.10.E04  **2011**mTn5STM.3.01.H03  **2011**mTn5STM.5.07.D02  **2011**mTn5STM.3.11.A08  **2011**mTn5STM.1.14.F07 | 498225-498224  499917-499916  498845-498846  498854-498853  497793-497794  500111-500110  498293-498292 |
| **SMc01373**  (1409085-1407793) | Putative DNA polymerase IV 1, Extends stalled forks: **(*dinB1*)** | **2011**mTn5STM.1.02.D07  **2011**mTn5STM.4.10.F04 | 1408298-1408299  1408427-1408426 |
| **SMa2355**  (1327042-1328151) | Nucleotidyltransferase/DNA polymerase | **2011**mTn5STM.4.05.D10 | 1327889-1327890 |
| **SMb21448**  (1378745-1379383) | Putative DNA polymerase | **2011**mTn5STM.3.11.F08 | 1379151-1379150 |
| **SMc01419**  (2267073- 2267624) | RNA polymerase sigma factor: **(*rpoE1*)** | **2011**mTn5STM.5.08.D04  **2011**mTn5STM.3.07.G07 | 2267397-2267396  2267443-2267444 |
| **SMc01506**  (2624014- 2623463) | RNA polymerasaesigma-E factor (sigma-24): **(*rpoE2*)** | **2011**mTn5STM.1.04.C05  **2011**mTn5STM.3.12.B10 | 2623877-2623878  2623780-2623779 |
| **SMc04051**  (3055740- 3055207) | RNA polymerase sigma-E factor (sigma-24): **(*rpoE4*)** | **2011**mTn5STM.1.13.E03 | 3055308-3055309 |
| **SMc03873**  (3539345- 3538509) | RNA polymerase sigma factor: **(*rpoH2 sigC*)** | **2011**mTn5STM.4.09.D11 | 3538958-3538959 |
| **Nucleases** |  |  |  |
| **SMc01336**  (1444408-1441634) | Probable ribonuclease E (RNAseE): **(*rne*)** | **2011**mTn5STM.4.07.G10 | 1442384-1442383 |
| **SMc02327**  (660513-660004) | Degrade RNA from RNA/DNA hybrids (RNAse H1): **(*rnhA2*)** | **2011**mTn5STM.1.13.B02 | 660227-660228 |
| **SMc00867**  (920230-919580) | Degrade RNA from RNA/DNA hybrids (RNAse H2): **(*rnhB*)** | SmPI_**1021**.12.04C9 | 919972-919971 |
| **SMc02760**  (38065-34496) | ssDNA exonuclease ATP-dep.: **(*recB*)** | **2011**mTn5STM.4.10.G03 | 36777-36776 |
| **SMb20689**  (1492447-1493220) | 3’-5’dsDNA exonuclease: **(*xthA4*)** | **2011**mTn5STM.1.13.G07  SmPI_**1021**.12.08C5 | 1492508-1492509  1492693-1492694 |
| **SMa0934**  (515693-520312) | Conjugal transfer protein. Identity  with ssDNA exonuclease: **(*recD*)** | SmPI_**1021**.12.06E7 | 519062-519063 |
| **SMc02843**  (192909- 193697) | Endonuclease III: **(*nth*)** | SmPI_**1021**.12.01A5  **2011**mTn5STM.1.02.E08 | 193210- 193211  193450- 193451 |
| **SMc00378**  (331801- 333378) | Exodeoxyribonuclease: **(*xseA*)** | **2011**mTn5STM.3.06.H09 | 332097- 332096 |
| **SMc00606**  (1277704- 1276949) | Ribonuclease | **2011**mTn5STM.4.09.C03 | 1277434-1277435 |
| **SMc01929**  (1394904-1396571) | Ribonuclease J: **(*rnj*)** | **2011**mTn5STM.3.10.C03  **2011**mTn5STM.3.13.E11 | 1394989-1394990  1395054-1395055 |
| **SMc00622**  (1297874- 1296726) | Ribonuclease D: **(*rnd1*)** | **2011**mTn5STM.4.07.H06 | 1297771-1297770 |
| **SMc04231**  (2055780- 2058734) | Excinuclease ABC subunit B: **(*uvrB*)** | **2011**mTn5STM.2.08.A01  **2011**mTn5STM.4.03.D11 | 2055881-2055880  2057901-2057900 |
| **Chaperones** |  |  |  |
| **SMc02857**  (209764-211686) | Heat shock proteins 70 (HSP70) chaperone: **(*dnaK*)** | **2011**mTn5STM.3.05.F08 | 211305- 211306 |
| **SMc00003**  (985233- 986288) | Molecular chaperone: **(*dna*J)** | SmPI_**1021**.12.01B9  **2011**mTn5STM.3.04.G09 | 985333- 985334  985818- 985819 |
| **SMc00913**  (870772- 869138) | 60 KD chaperonin A: **(*groEL1*)** | **2011**mTn5STM.1.08.G04  SmPI_**1021**.12.04B10  **2011**mTn5STM.2.08.H06 | 870554- 870555  870320- 870319  870019- 870020 |
| **SMc01758**  (1305463- 1303838 | 60 KD chaperonin B: **(*groEL4*)** | **2011**mTn5STM.3.03.B05  **2011**mTn5STM.2.05.F01  **2011**mTn5STM.1.03.G02 | 1304928-1304927  1304421-1304420  1304205-1304206 |
| **SMc04456**  (2527640- 2527302) | Chaperone: **(*csaA*)** | **2011**mTn5STM.2.09.D09 | 2527480-2527479 |
| **SMa0124**  (67826- 66195) | Chaperonin: **(*groEL3*)** | **2011**mTn5STM.1.04.A01  **2011**mTn5STM.1.05.H11 | 66337- 66336  66228- 66229 |
| **SMb21183**  (932442- 930556) | Probable chaperonine, heat shock hsp90 proteins family: **(*htpG*)** | **2011**mTn5STM.2.12.G12 | 931519- 931518 |
| **Other functions** |  |  |  |
| **SMc00321**  (273485- 274417) | tRNA pseudouridine sintase B: **(*truB*)** | **2011**mTn5STM.4.10.C12  **2011**mTn5STM.2.08.G08  **2011**mTn5STM.4.11.B07 | 273584- 273583  273737- 273736  274206- 274205 |
| **SMc02693**  (2543265- 2543981) | Peptidyl-tRNA hydrolase: **(*pth*)** | **2011**mTn5STM.2.07.H02 | 2543849-2543850 |
| **SMc00713**  (2867621- 2867070) | Cation transport: **(*chaC*)** | **2011**mTn5STM.1.11.B04  SmPI_**1021**.12.05E3 | 2867465-2867466  2867447-2867446 |
| **SMc00399**  (349712- 348726) | Probable Mg. and Co. transport transmembrane protein: **(*corA1*)** | **2011**mTn5STM.2.03.D08  **2011**mTn5STM.5.03.D01 | 348992- 348993  348721- 348722 |
| **SMc00874**  (911877- 912854) | Probable Mg. and Co. transport transmembrane protein: **(*corA2*)** | **2011**mTn5STM.2.08.F04 | 912221- 912222 |
| **SMc00697**  (2883543- 2882239) | Hypothetical transmembrane protein: **(*corB****)* | **2011**mTn5STM.1.09.E10  **2011**mTn5STM.4.09.G12 | 2883216-2883217  2882463-2882464 |
| **SMc01261**  (1514121- 1515542) | Putative transporter transmembrane protein: **(*mgtE*)** | Deleted and substituted by  Spectinomicyn resistance cassette in *S.m.* **2011** | 1514121- 1515542 |
| **SMc01023**  (1548277- 1549044) | Triosephosphate isomerase: **(*tpiA1*)** | **2011**mTn5STM.4.08.B01 | 1548617-1548616 |
| **SMc03979**  (2975447- 2976454) | Glyceraldehyde 3-phosphate  dehydrogenase: **(*gap*)** | **2011**mTn5STM.2.11.H09 | 2975948-2975947 |
| **SMc03981**  (2977053- 2978252) | Probable phosphoglycerate kinase: **(*pgk*)** | **2011**mTn5STM.1.09.G04  **2011**mTn5STM.3.08.F07 | 2977533-2977534  2977789 2977790 |
| **SMc03978**  (2973395- 2975377) | Probable transketolase: **(*tkt2*)** | **2011**mTn5STM.1.12.D08 | 2974676-2974675 |
| **SMc02163**  (532370- 530748) | Glucose-6-phosphate isomerase: (***pgi*)** | **2011**mTn5STM.4.12.B11 | 531127- 531126 |
| **SMb21579**  (1119502- 1119945) | Putative transcriptional regulator: **(*hmrR2***) | **2011**mTn5STM.4.11.A09  SmPI_**1021**.12.07H8 | 1119529-1119530  1119609-1119610 |
| **SMc00109**  (1072051-1071584) | Putative transcriptional regulator | **2011**mTn5STM.1.05.G08  **2011**mTn5STM.3.07.E03 | 1071836-1071835  1071790-1071791 |
| **SMc02659**  (1164419-1166644) | Putative GTP pyrophosphokinase: **(*relA*)** | **2011**mTn5STM.2.12.C04 | 1165740-1165741 |
| **SMc02496**  (3298640-3296436) | Probable primosomal protein N' (replication factor Y): **(*priA*)** | SmPI_**1021**.12.03F10 | 3297084-3297083 |
| **SMc00339**  (296513-294516) | Adenylate cyclase 1 protein: **(*cyaA*)** | **2011**mTn5STM.4.08.H11  **2011**mTn5STM.1.14.A01 | 296298-296299  295320-295319 |
| **SMb21488**  (1409489-1411498) | Putative cytochrome o ubiquinol oxidase chain I protein: **(*cyoB*)** | **2011**mTn5STM.4.03.B02  **2011**mTn5STM.4.06.A01 | 1410055-1410054  1411114-1411115 |
