# Supplementary material for: Host Factors Influencing the Retrohoming Pathway of Group II Intron RmInt1, Which Has an Intron-Encoded Protein Naturally Devoid of Endonuclease Activity
Source: PLoS One. 2016 Sep 2;11(9):e0162275. doi: 10.1371/journal.pone.0162275 (PMC5010178; doi:10.1371/journal.pone.0162275)
Supplement: S2 Table — In the first column are listed the wild type strains used in this study, the loci and the genome coordinates, according to GenDB browser (http://www.cebitec.uni-bielefeld.de/CeBiTec/rhizogate). The second column indicates the probable function of the genes. The third column corresponds to the mutant used to perform the retrohoming assay. The fourth column indicates the position of the insertion of the mTn5 in 2011 mutants or the plasmid in 1021 mutants. The fifth column shows the retrohoming efficiency (% ± S.E.M.).The last column indicates the homing efficiency relativized to the wild type (S. meliloti 2011 or 1021). (DOCX) [file pone.0162275.s003.docx]

**S2 Table**

| ***S. meliloti* WT strains** |  |  |  | **Homing**  **(% ± S.E.M.)** | **% Homing**  **(Rel. WT)** |
| --- | --- | --- | --- | --- | --- |
| **2011** |  |  |  | 23.28 ± 1.22 | 100 |
| **1021** |  |  |  | 19.9 **±** 3.69 | 100 |
|  |  |  |  |  |  |
| **Locus**  **(Genome position)** | **Probable Function (*gene*)** | **Mutant** | **Mutation Position** | **Homing**  **(% ± S.E.M.)** | **% Homing**  **(Rel. WT)** |
| **Helicases** |  |  |  |  |  |
| **SMc01461**  (2306850-2309423) | 3’-5’ RNA/DNA hybrids helicase: (***uvrD1***) | **2011**mTn5STM.2.03.F05 | 2307492-2307493 | 27.13 **±** 5.81 | 116.53 |
| **SMc01566**  (2508771-2506861) | ATP-dep 3’-5’ DNA helicase: **(*recQ***) | **2011**mTn5STM.3.02.G02 | 2507083-2507082 | 24.19 ± 8.49 | 103.9 |
| **Repair protein** |  |  |  |  |  |
| **SMc00556**  (1227431- 1225971) | DNA repair protein: **(*radA*)** | **2011**mTn5STM.2.02.C03 **2011**mTn5STM.4.04.E12 | 1226930- 226931 1226787-1226788 | 46.19 ± 6.7  48.67 ± 12.5 | 198.41  209.06 |
| **Polymerase and related proteins.** |  |  |  |  |  |
| **SMc03788**  (3455860-3452510) | DNA polymerase III alpha chain  Major replicative enzyme  (DNA Pol III): **(*dna*E2)** | **2011**mTn5STM.4.13.F08 | 3452877-3452878 | 28.06 ± 1.94 | 120.53 |
| **SMc01373**  (1409085-1407793) | Putative DNA polymerase IV 1, Extends stalled forks: **(*dinB1*)** | **2011**mTn5STM.1.02.D07  **2011**mTn5STM.4.10.F04 | 1408298-1408299  1408427-1408426 | 27.12 ± 1.22  20.81 ± 8.66 | 116.49  89.39 |
| **SMa2355**  (1327042-1328151) | Nucleotidyltransferase/DNA polymerase | **2011**mTn5STM.4.05.D10 | 1327889-1327890 | 20.15 ± 4.94 | 86.55 |
| **SMc01419**  (2267073- 2267624) | RNA polymerase sigma factor: **(*rpoE1*)** | **2011**mTn5STM.5.08.D04 | 2267397-2267396 | 26.39 ± 3.6 | 113.35 |
| **Nucleases** |  |  |  |  |  |
| **SMc01336**  (1444408-1441634) | Probable ribonuclease E (RNAseE): **(*rne*)** | **2011**mTn5STM.4.07.G10 | 1442384-1442383 | 35.72 ± 1.48 | 153.43 |
| **SMc00867**  (920230-919580) | Degrade RNA from RNA/DNA hybrids (RNAse H2): **(*rnhB*)** | SmPI_**1021**.12.04C9 | 919972-919971 | 34.34 ± 1.48 | 172.56 |
| **SMc02760**  (38065-34496) | ssDNA exonuclease ATP-dep.: **(*recB*)** | **2011**mTn5STM.4.10.G03 | 36777-36776 | 30.74 ± 7.62 | 132.04 |
| **SMb20689**  (1492447-1493220) | 3’-5’dsDNA exonuclease: **(*xthA4*)** | **2011**mTn5STM.1.13.G07  SmPI_**1021**.12.08C5 | 1492508-1492509  1492693-1492694 | 17.56 ± 1.79  5.36 ± 5.36 | 75.42  26.93 |
| **SMa0934**  (515693-520312) | Conjugal transfer protein. Identity  with ssDNA exonuclease TrA1: **(*recD*)** | SmPI_**1021**.12.06E7 | 519062-519063 | 21.89 ± 3.06 | 110 |
| **Chaperones** |  |  |  |  |  |
| **SMc02857**  (209764-211686) | Heat shock proteins 70 (HSP70) chaperone: **(*dnaK*)** | **2011**mTn5STM.3.05.F08 | 211305- 211306 | 33.35 ± 1.61 | 143.25 |
| **Other functions** |  |  |  |  |  |
| **SMc00399**  (349712- 348726) | Probable Mg. and Co. transport transmembrane protein: **(*corA1*)** | **2011**mTn5STM.2.03.D08 | 348992- 348993 | 41.28 ± 6.06 | 177.31 |
